# Differential Assessment of Factor Xa Activity and Global Blood Coagulability Utilizing Novel Dielectric Coagulometry

**DOI:** 10.1038/s41598-018-34229-6

**Published:** 2018-10-31

**Authors:** Satomi Hamada, Yuki Hasegawa, Ai Oono, Anna Suzuki, Naomi Takahashi, Takuro Nishimura, Takatoshi Koyama, Michio Hagihara, Shuji Tohda, Tetsushi Furukawa, Kenzo Hirao, Tetsuo Sasano

**Affiliations:** 1 Department of Biofunctional Informatics, Tokyo Medical and Dental University (TMDU), Tokyo, Japan; 2 Department of Clinical Laboratory, Medical Hospital, Tokyo Medical and Dental University (TMDU), Tokyo, Japan; 3 Heart Rhythm Centre, Tokyo Medical and Dental University (TMDU), Tokyo, Japan; 4 Department of Laboratory Molecular Genetics of Haematology, Tokyo Medical and Dental University (TMDU), Tokyo, Japan; 5 Department of Bio-informational Pharmacology, Medical Research Institute, Tokyo Medical and Dental University (TMDU), Tokyo, Japan; 6 Department of Cardiovascular Medicine, Tokyo Medical and Dental University (TMDU), Tokyo, Japan; 7 Department of Cardiovascular Physiology, Tokyo Medical and Dental University (TMDU), Tokyo, Japan

## Abstract

An easy-to-use assessment for activated factor X (FXa) is lacking despite its pivotal role in the coagulation. Dielectric blood coagulometry (DBCM) was recently invented as a novel assessment tool for determining the whole blood coagulability by measuring the temporal change in the permittivity of blood. We previously reported that it could evaluate the global blood coagulability. This study aimed to apply the DBCM for assessing FXa activity and its inhibition by anticoagulants. We performed the DBCM analysis along with measurement of the FXa activity by a fluorometric assay in samples from healthy subjects, and identified a new index named maximum acceleration time (MAT) that had a correlation to the FXa activity. Next the DBCM analysis was performed using blood samples mixed with anticoagulants (unfractionated heparin, dalteparin, and edoxaban). Blood samples with three anticoagulants had different profiles of the temporal change in the permittivity, reflecting their different selectivity for FXa. We compared the MAT with the anti-FXa activity assay, and found that the prolongation of MAT was similarly correlated with the anti-FXa activity regardless of the type of anticoagulants. In conclusion, the DBCM has the possibility for evaluating the innate FXa activity and effect of anticoagulants focusing on their FXa inhibition.

## Introduction

Thrombosis is a common pathophysiological condition underlying strokes, ischemic heart disease, peripheral arterial disease, and deep vein thrombosis. Thrombi are generated as a final product of the coagulation cascade. The reaction of the coagulation cascade is initiated by intrinsic or extrinsic pathways, followed by the activation of a common pathway. In a conventional coagulation cascade model, activated factor X (FXa) composes the initial part of the common pathway, and FXa cleaves prothrombin to convert it into thrombin. Thrombin converts fibrinogen into fibrin, resulting in the blood clot formation^1^. The alternative cell-based model of coagulation indicates that a prothrombinase complex comprised of FXa is critical for the amplification of thrombin^2^. Both models suggest that FXa plays a pivotal role in the upper stream of thrombin generation.

Since FXa plays a major role in the coagulation cascade, several anticoagulants targeting FXa have been developed. Unfractionated heparin (UFH) has been mostly utilized in clinical settings, inhibiting thrombin and FXa, accompanied with antithrombin. Low molecular weight heparin (LMWH) later came into the market, and is characterized by higher selectivity for FXa inhibition than UFH. Vitamin K antagonists also inhibit FXa and thrombin with other coagulation factors, VII and IX. Recently, the usage of direct FXa inhibitors (FXaIs) has been rapidly expanding for the prevention of strokes related to atrial fibrillation, and for deep vein thrombosis^3–5^.

Given that the usage of more selective FXa inhibitors has expanded, it becomes more important to assess FXa activity in relation to the blood coagulability. However, the quantitative assessment of FXa activity has been limited by a lack of easy-to-use assays. Despite recent advancements in the development of whole blood coagulation assays^6^, their application to quantify FXa activity has not been established.

Recently a novel dielectric blood coagulometry (DBCM) was invented for the evaluation of whole blood coagulability^7,8^. DBCM measures the temporal change in whole blood dielectric permittivity, which mainly represents the aggregation and deformation of red blood cells. We previously reported that the novel index, the end of acceleration time (EAT), represented the whole blood coagulability using the DBCM, and the EAT had the potential to evaluate small changes in the hypercoagulable state^9^. Since DBCM detects the temporal change in the dielectric permittivity in relation to the activation of the coagulation cascade, we hypothesized that the intermediate step of the permittivity change might represent the upper stream of thrombin generation, including FXa activation, and DBCM might be able to evaluate FXa activity separately with global blood coagulation. In this study, we tried to establish an index representing FXa activity, and studied its relevance using blood samples with the administration of nonselective and selective FXa inhibitors.

## Results

### Subjects included in this study

Seventy-four apparently healthy subjects (27 ± 8 years old, 28 males) were enrolled in this study. We confirmed they had no medical history, no medications, no family history of coagulation deficiency, and no abnormal bleeding events. We examined the basic characteristics of 10 subjects, and found all of the parameters were within normal range (Supplementary Table S1).

### Identification of the DBCM parameters representing the FXa activity

**Figure 1:**
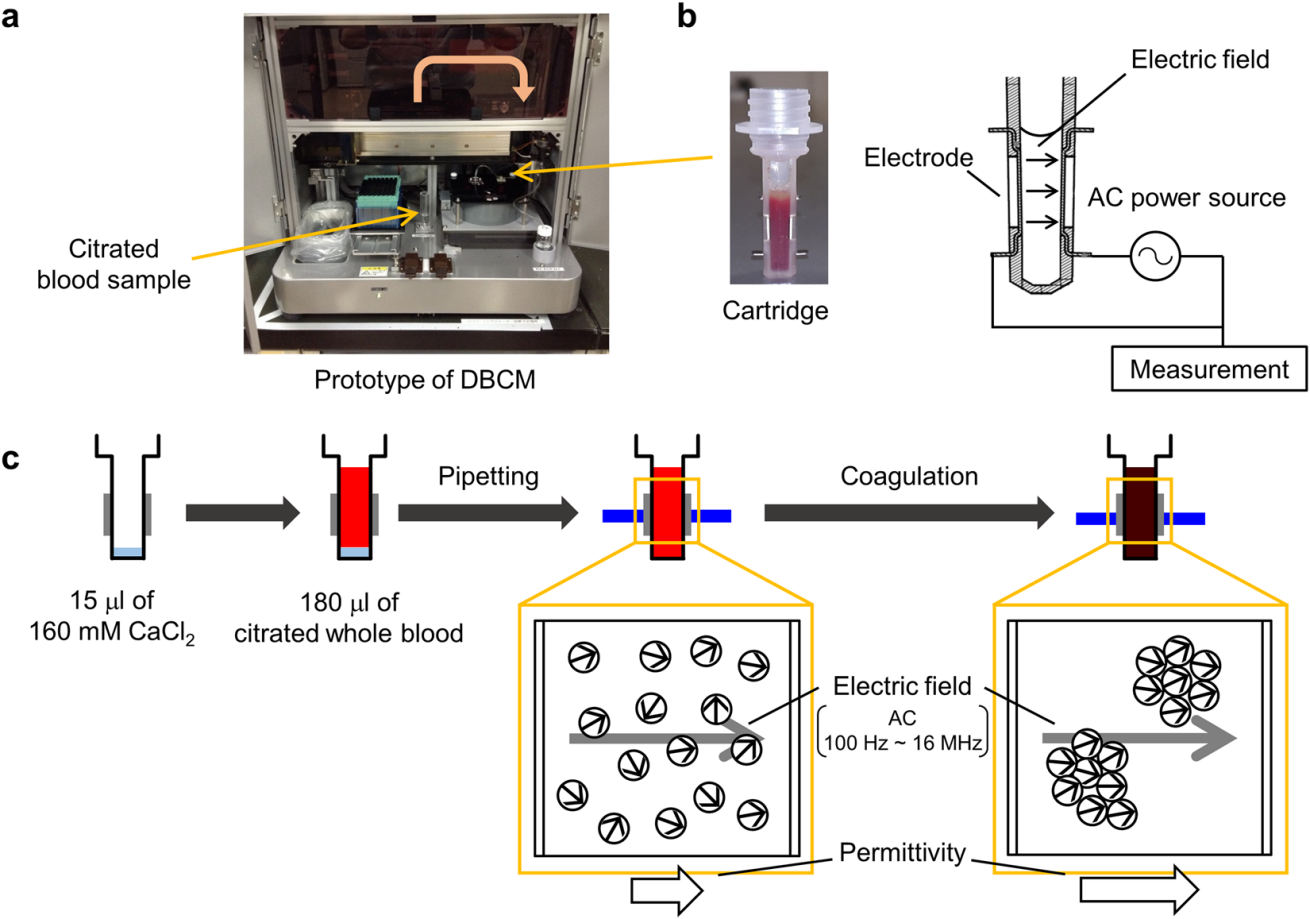
Experimental setup of the DBCM. (**a**) The general assembly of the prototype of DBCM. Blood sample tube is placed at the holder, and 180 μl of citrated blood is drawn and poured into a disposable cartridge. (**b**) The cartridge consists of an insulator and 2 electrodes. During a measurement, AC with a frequency of 100 to 16 MHz is applied, and the dielectric permittivity is continuously measured. (**c**) Recalcification induces the coagulation of citrated whole blood. The dielectric permittivity increases reflecting the progression of coagulation. DBCM, dielectric blood coagulometry; AC, alternating current.

**Figure 2:**
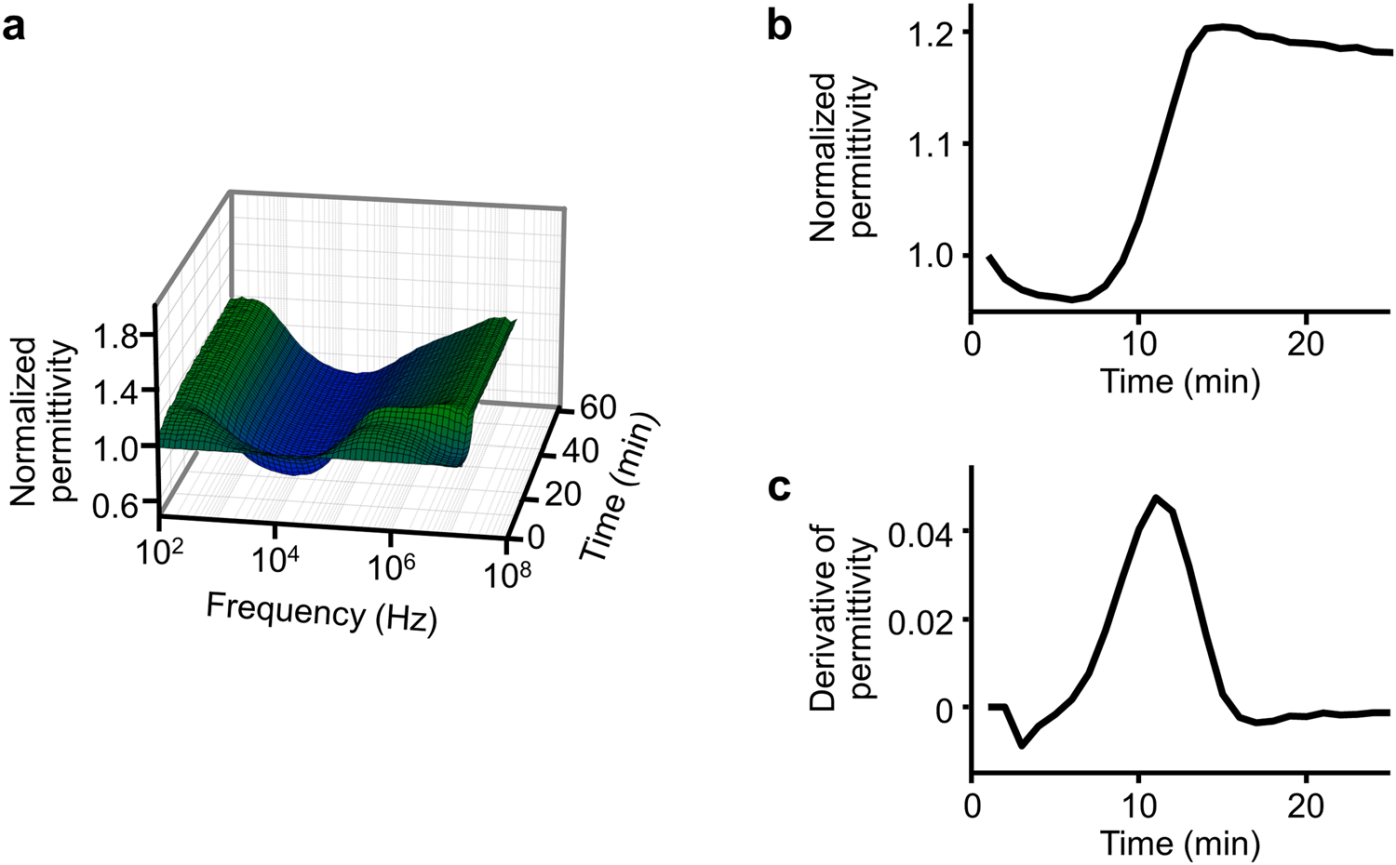
Representative traces in the dielectric permittivity change. (**a**) The dielectric permittivity normalized by its initial value is plotted against the frequency of an alternative current and the time after recalcification. (**b**) The temporal change in the permittivity at 10 MHz shows a sigmoidal increase. (**c**) The derivative of the temporal change in the permittivity exhibits a unimodal curve.

First, we studied whether the DBCM could be used to evaluate FXa activity. The DBCM analysis was performed and compared with the fluorometric assay for the FXa activity in 30 healthy subjects. The prototype of DBCM used in this study measured the dielectric permittivity in a frequency range from 100 Hz to 16 MHz every 1 minute after recalcification (Figs 1 and 2a). Since previous reports have shown that the permittivity change at 10 MHz represents the whole blood coagulation^8^, we used the permittivity change at 10 MHz (Fig. 2b) and calculated the derivative as previously described^9^. The derivative curve exhibited a single peak pattern (Fig. 2c).

**Figure 3:**
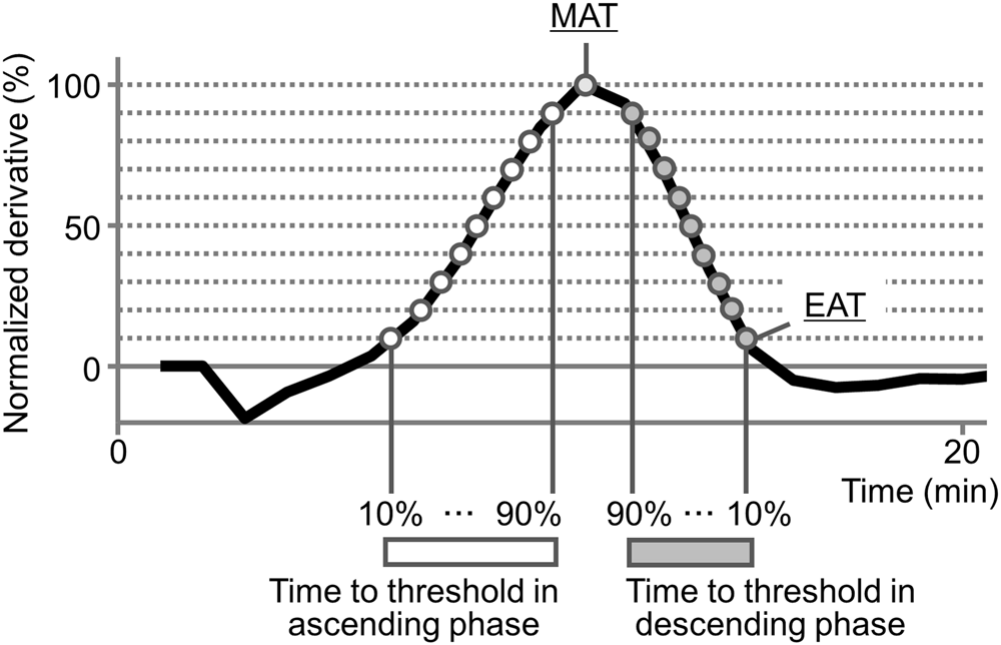
Definition of the DBCM parameters. The threshold lines are set from 10% to 100% of the maximum value with 10% steps (dotted line), and the times to thresholds are measured in the ascending phase (open circle) and descending phase (grey circle). The time to peak is named the maximum acceleration time (MAT), and the time to 10% of the maximum value in the descending phase is named the end of acceleration time (EAT).

Then we measured the time to threshold, by scanning the threshold line from 10% to 90% of the maximum value of the derivative (Fig. 3). Thus, we obtained 9 values representing the time to each threshold in the ascending and descending phases, respectively. We also obtained the time to reach the maximum value. In a previous paper, we specifically named the time to cross 10% of the maximum value in the descending phase as the end of acceleration time (EAT), which reflected the blood coagulability. In this study, we newly defined the time to reach its maximum value as the maximum acceleration time (MAT).

**Figure 4:**
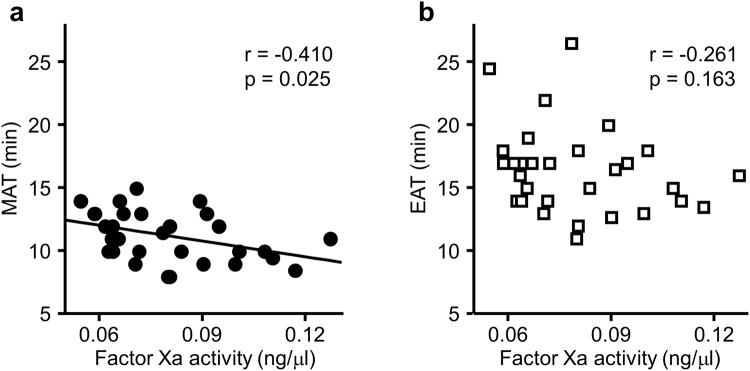
Correlation between the DBCM parameters and FXa activity. Scatter plots and single regression analyses between the fluorometric FXa activity and the MAT or the EAT. (**a**) The MAT shows a significant inverse correlation to the FXa activity. (**b**) No significant correlation is observed between the EAT and FXa activity. MAT, maximum acceleration time; EAT, end of acceleration time.

To clarify whether some of these values represented FXa activity, we evaluated the correlation between those times to threshold and FXa activity measured by a fluorometric assay. The regression analysis revealed that the FXa activity had an inverse correlation to the MAT and some of the times to threshold set in the ascending phase of the derivative curve. Among those, the MAT had the highest correlation coefficient (r = −0.410, p = 0.025) (Table 1, Fig. 4a). That result indicated that the MAT had the possibility of representing the innate FXa activity, at least in healthy subjects. The times to threshold set in the descending phase of the derivative curve, including the EAT, also had a tendency to have a negative correlation to the FXa activity. However, the correlation was too weak to reach statistical significance (Table 1, Fig. 4b). The descending phase of the derivative curve might be influenced not only by the FXa activity, but also by the downstream reaction in the coagulation cascade presumably including thrombin. To find a more appropriate parameter representing FXa activity, we calculated the time difference and the area under the curve based on the times to each threshold (Supplementary Fig. S1). However, none of them showed a significant correlation to the FXa activity (Supplementary Table S2).

**Table 1:** Correlations between the DBCM parameters and FXa activity.

DBCM parameter	r	p
Time to threshold in the ascending phase		
10% of maximum	−0.334	0.071
20% of maximum	−0.334	0.072
30% of maximum	−0.301	0.106
40% of maximum	−0.409	0.025*
50% of maximum	−0.374	0.042*
60% of maximum	−0.367	0.046*
70% of maximum	−0.380	0.038*
80% of maximum	−0.398	0.029*
90% of maximum	−0.338	0.068
Maximum acceleration time	−0.410	0.025*
Time to threshold in the descending phase		
90% of maximum	−0.309	0.097
80% of maximum	−0.324	0.081
70% of maximum	−0.295	0.113
60% of maximum	−0.308	0.098
50% of maximum	−0.285	0.127
40% of maximum	−0.291	0.119
30% of maximum	−0.265	0.157
20% of maximum	−0.264	0.158
10% of maximum	−0.261	0.163

DBCM, dielectric blood coagulometry.

*p < 0.05.

From these findings, we hypothesized that the MAT represented the FXa activity to some extent. Therefore, we focused on the characteristics of the MAT with the EAT, an already established index for whole blood coagulation.

### Different responses of the DBCM parameters with the administration of anticoagulants

Next, we examined whether the DBCM responded to the anticoagulants which had the different selectivity for FXa. We added 3 anticoagulants (UFH, LMWH, and FXaI) to the blood samples with serial concentrations, and performed the DBCM analyses. It is well known that UFH has the lowest selectivity for FXa. LMWH has higher selectivity for FXa over thrombin compared to UFH, and FXaI has the highest selectivity for FXa among 3 anticoagulants.

UFH and LMWH were added to the blood samples with serial concentrations (0, 0.05, 0.10, and 0.15 U/ml or IU/ml, respectively), and FXaI was also added to the blood samples with serial concentrations (0, 100, 200, and 400 ng/ml). We chose the concentration range of LMWH and FXaI according to the effective plasma concentration in clinical settings, using data obtained from the drug information of LMWH (dalteparin, Fragmin, Pfizer) and FXaI (edoxaban, Lixiana, Daiichi-sankyo). The concentration of UFH was adjusted to that of LMWH.

**Figure 5:**
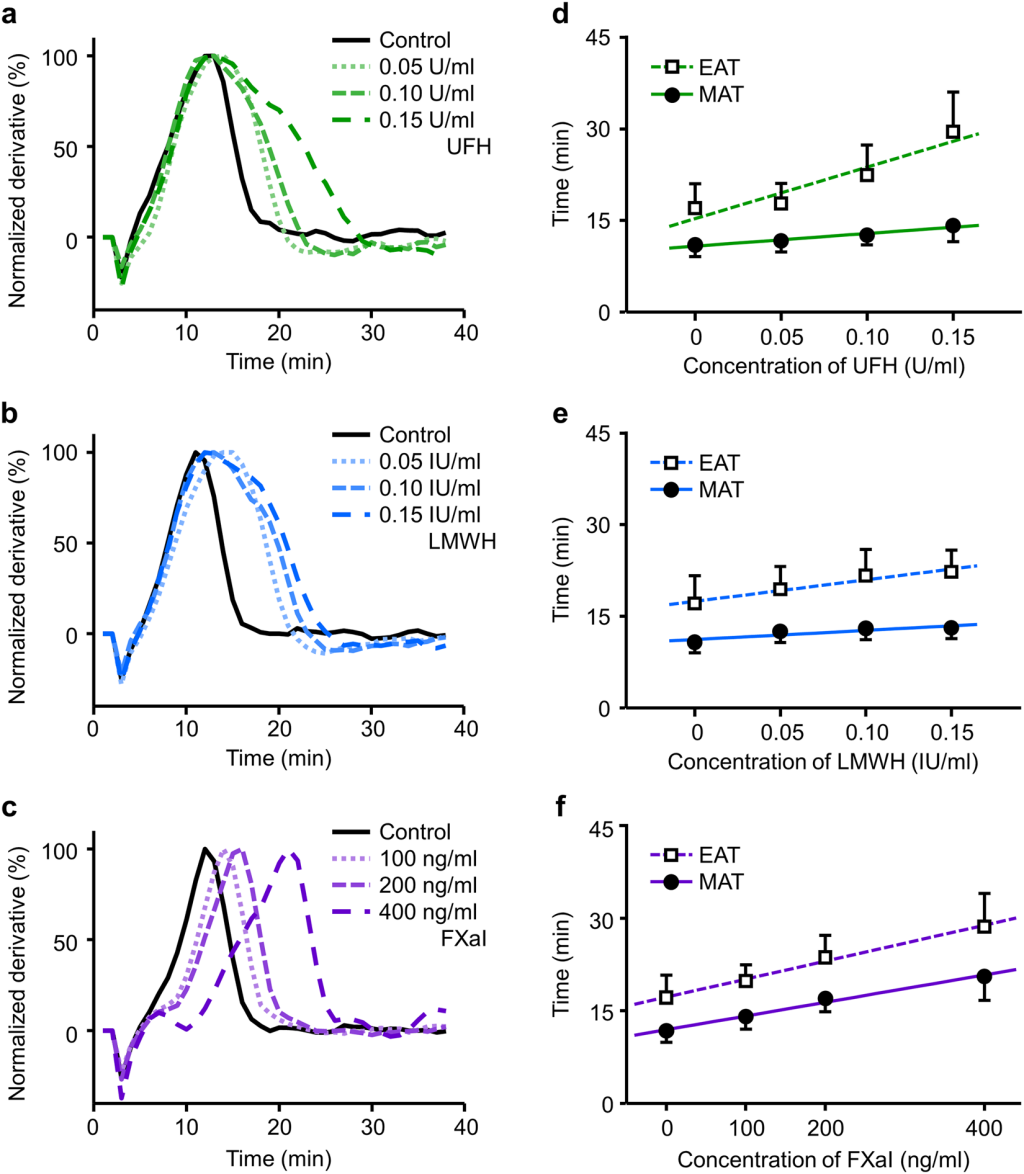
Change in the normalized derivative curve of the permittivity and DBCM parameters in response to 3 anticoagulants. (**a**–**c**) Representative curves of the normalized derivative of the permittivity in samples mixed with serial concentrations of UFH (**a**), LMWH (**b**), and FXaI (**c**). (**d**–**f**) The MAT, EAT and their regression lines in samples with serial concentrations of UFH (**d**), LMWH (**e**), and FXaI (**f**). The MAT and EAT are expressed as mean − or +standard deviation. UFH, unfractionated heparin; LMWH, low molecular weight heparin; FXaI, factor Xa inhibitor; the other abbreviations are the same as in Fig. 4.

After an administration of anticoagulants, the normalized derivative curve of the permittivity shifted to the right, indicating the prolongation of coagulation, in a dose-dependent manner (Fig. 5a–c). The shapes of the curves differently changed among 3 anticoagulants: UFH mainly prolonged the descending phase, FXaI mainly prolonged the ascending phase, and the effect of LMWH was the middle. We summarized the prolongation of MAT and EAT against the concentration of anticoagulants in Fig. 5d–f. Although both the MAT and EAT showed dose-dependent prolongation with the addition of all anticoagulants, their effects on the EAT and MAT were different.

**Figure 6:**
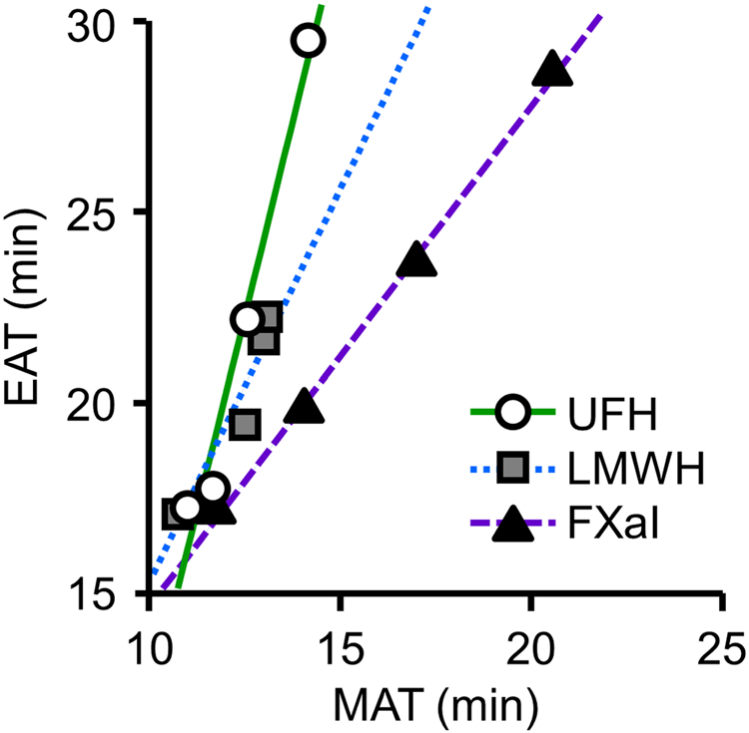
Relationships between the MAT and EAT in response to anticoagulants with different FXa selectivity. The EAT is plotted as a function of the MAT in samples with serial concentrations of 3 anticoagulants (UFH, LMWH, and FXaI). An anticoagulant that had a higher FXa selectivity exhibited a smaller regression slope, indicating a more prominent effect for the MAT than EAT. The abbreviations are the same as in Fig. 5.

Thus we compared the prolongation of the MAT and EAT in response to the administration of UFH, LMWH, and FXaI (Fig. 6). The average values of the MAT and EAT in the blood samples with serial concentrations of anticoagulants were plotted, and single regression analyses were performed. The slope of regression line was the largest with UFH, next with LMWH, and the smallest with FXaI (4.07 [95% confidence interval, 1.91–6.23] for UFH, 2.07 [0.08–4.06] for LMWH, and 1.31 [1.15–1.48] for FXaI). The higher FXa selectivity the anticoagulant had, the larger the percentage of the MAT in the prolongation became.

**Figure 7:**
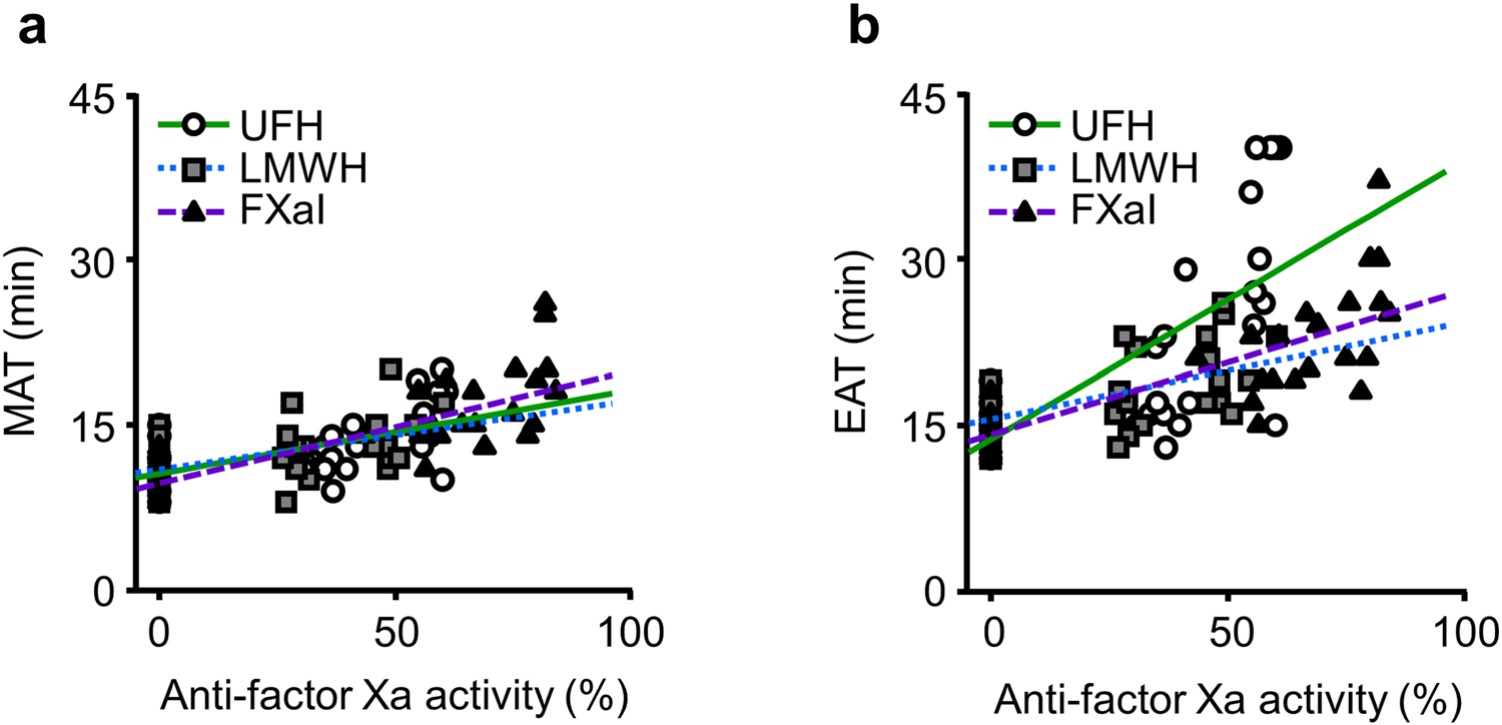
Relationships between the DBCM parameters and anti-FXa activity in samples mixed with anticoagulants. The MAT (**a**) and EAT (**b**) are plotted against the anti-FXa activity in samples mixed with serial concentrations of UFH, LMWH, and FXaI. The anti-FXa activity was expressed as relative value to its measurement range. The regression lines for all 3 anticoagulants are similar in the MAT, but not in the EAT. The abbreviations are the same as in Fig. 5.

We then measured the anti-FXa activity and compared it with the DBCM parameters in blood samples with 3 anticoagulants (Fig. 7). The concentration of the anticoagulants used in this comparison were 0, 0.075, and 0.15 U/ml in UFH, 0, 0.075, and 0.15 IU/ml in LMWH, and 0, 200, and 400 ng/ml in FXaI. We expressed the anti-FXa activity as a percentage of its maximum value of measurement range in the reagents. The MAT and EAT showed significant positive correlations to anti-FXa activity in all samples. Of interest, the regression lines between the MAT and anti-FXa activity were almost the same among all anticoagulants, but those between the EAT and anti-FXa activity showed different slopes. This finding indicated that the MAT was able to assess the reduction in FXa activity regardless of types of anticoagulants.

We also assessed the correlation of the activated partial thromboplastin time (APTT) to the MAT or EAT in samples with each anticoagulant, because APTT is routinely used for monitoring the effect of UFH (Supplementary Fig. S2). Although the MAT and EAT had positive correlations to the APTT, their regression lines were different among the anticoagulants. Thus we speculate the EAT and MAT might work as independent and novel parameters to assess blood coagulability.

## Discussion

In this study, we established a new index, the maximum acceleration time (MAT), using the DBCM analysis. We speculated that the MAT represented the FXa activity based on 3 findings. First, the MAT showed a significant inverse correlation to the FXa activity measured using a fluorometric assay in healthy subjects. Second, the prolongation of the MAT in comparison to that of the EAT was larger with the administration of anticoagulants with higher selectivity for FXa. Third, the slopes of regression lines between the MAT and anti-FXa activity were consistent in all type of anticoagulants. This report showed that the DBCM had the potential for evaluating the innate FXa activity and anti-FXa activity by anticoagulants.

**Figure 8:**
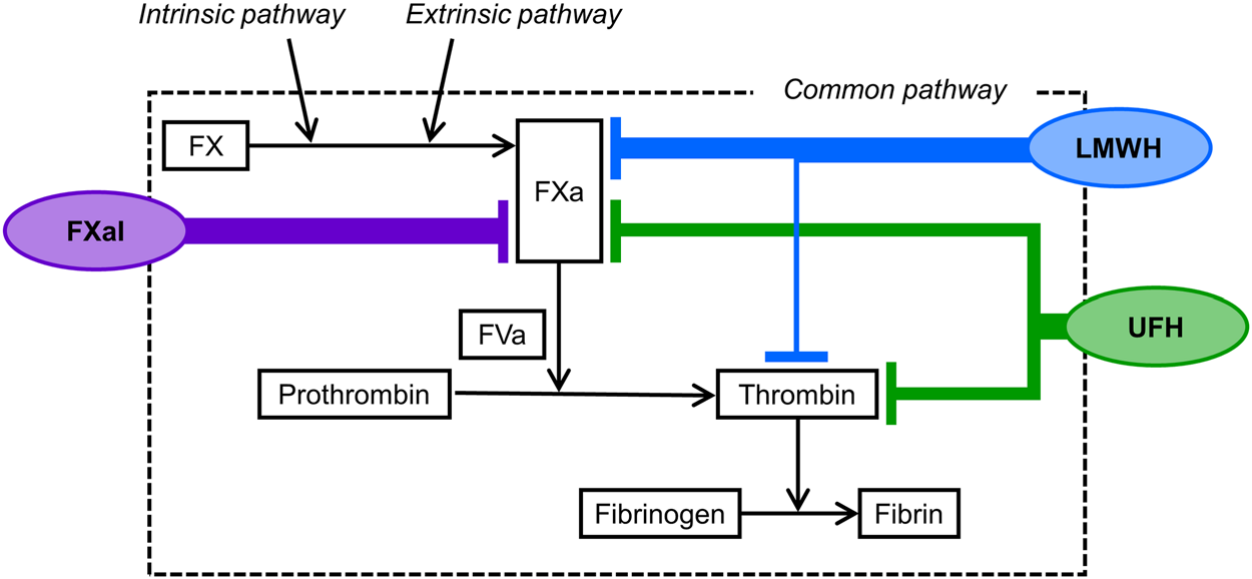
Coagulation cascade and the anticoagulants used in this study. Coagulation cascade starts with intrinsic or extrinsic pathways. Both pathways convert factor X (FX) into activated factor X (FXa). FXa converts prothrombin into thrombin with the presence of activated factor V (FVa). Anticoagulants utilized in this study inhibit the activity of FXa and thrombin with different selectivity: UFH inhibits FXa and thrombin similarly, LMWH supresses FXa more than thrombin, and FXaI is a selective blocker of FXa. The abbreviations are the same as in Fig. 5.

In both the classical coagulation cascade model and cell-based model, FXa plays a critical role in the generation of thrombin^1,2^ and further downstream of the blood clot formation (Fig. 8). Although the coagulation cascade has complex feedback mechanisms, factor X activation substantially precedes the activation of thrombin. We hypothesized that the contribution of the FXa activity to the DBCM parameters changed time-dependently, and sought the adequate time point to represent the FXa activity. When we scanned the threshold with 10% steps of the maximum value of the derivative of permittivity, the times to reach thresholds from 40% to 80% in the ascending phase and the MAT had a significant inverse correlation to the FXa activity. Thus, the former half of the derivative of permittivity curve might be related to the FXa activity. We selected the MAT as an optimal index for FXa activity because the MAT had the largest correlation coefficient. However, further large-scale analyses may change the definition of the parameter to indicate FXa activity.

In samples mixed with UFH, LMWH (dalteparin), and FXaI (edoxaban), the MAT was prolonged dose-dependently. These anticoagulants had the different selectivity for FXa inhibition: UFH inhibits FXa and thrombin similarly, dalteparin suppresses FXa more than thrombin^10^, and edoxaban is known as a selective blocker of FXa^11^ (Fig. 8). The prolongation of the MAT was significantly correlated with the anti-FXa activity, and the slope of the regression line was similar regardless of types of anticoagulants. On the other hand, the EAT showed different prolongation rate against the anti-FXa activity. In addition, the prolongation of the MAT compared to that of the EAT became greater with the administration of anticoagulants with higher selectivity for FXa. Therefore, we considered that the MAT was also specific for FXa activity in samples with anticoagulants.

Recently the use of anticoagulants with high FXa selectivity has expanded. They are administered with fixed doses or occasionally in adjusted doses according to body weight or renal function, without laboratory monitoring. However, monitoring is useful in several situations such as in patients who have bleeding, thromboembolic events, invasive procedures, extremes of body weight, renal dysfunction, suspected non-compliance, or overdoses^12,13^. At the present time, the measurement of the plasma concentration of drugs by liquid chromatography-tandem mass-spectrometry/mass-spectrometry is used for the monitoring of direct FXaIs^14–16^. Anti-FXa activity assay is recommended for the monitoring of LMWHs^12^ and also used for the monitoring of direct FXaIs^17,18^. These assays are relatively expensive and require special calibrators and particular expertise^13,19^. We considered DBCM could be one of the monitoring tools for anticoagulants with FXa inhibition using MAT, as with several assays which have been reported to respond to them^20–23^.

In contrast to the MAT, we speculated that the EAT represented the global blood coagulability including the activity of FXa and downstream molecules. The EAT had no significant correlation to the FXa activity, together with other parameters calculated using thresholds in the descending phase of the derivative curve of the permittivity change. This finding suggested that the EAT was not specific for FXa activity.

We previously showed that the EAT was prolonged dose-dependently with the addition of UFH, and that it correlated strongly with the APTT^9^. We confirmed them in samples with LMWH and FXaI in this study. However, the regression line of the EAT for APTT in the blood samples mixed with FXaI was relatively different from those with UFH and LMWH. Thus, the interchangeability between the EAT and APTT might not be consistent depending on anticoagulants.

DBCM uses whole blood and continuously measures the change in the permittivity during the clot formation. This resembles thromboelastgraphy (TEG) and rotational thromboelastmetry (ROTEM), point-of-care tests which measure the change in the viscoelasticity^24^. They are known to measure the coagulation profiles from the initiation to propagation phases^25,26^, and have been reported to respond to LMWHs^20,21^ and direct FXaIs^22,23^. We thought the positioning of DBCM in coagulation assays is in line with TEG/ROTEM, though further study is required to compare the change in the permittivity and viscoelasticity to clarify the difference between DBCM and TEG/ROTEM.

The MAT and EAT exhibited different responses to the anticoagulants depending on their selectivity for FXa, thus we believed that DBCM had the potential to differentially evaluate the FXa activity and global blood coagulability. This characteristic has not been reported with other coagulation assays using whole blood. With ROTEM, there was no difference noted in the response to thrombin inhibitors and FXa inhibitors^27^. Regarding other coagulation assays using plasma, thrombin generation assay (TGA), a method to measure the generation of thrombin continuously using a chromogenic substrate, is known to exhibit different responses between thrombin and FXa inhibitors^28,29^. DBCM may be able to monitor the change in the coagulation factors like TGA, though we need further investigation to assess the clinical utility of the detailed evaluation.

This study had several limitations. First, since this was a proof of concept study, the number of subjects was relatively small. Second, we did not evaluate the effects of other LMWHs, direct FXaIs, or thrombin inhibitors. We need further investigation to confirm the relationship between the DBCM parameters and these drugs in the future. Third, we did not study the relationship between the DBCM parameters and clinical outcome. It should be elucidated whether the DBCM parameters can predict ischemic strokes or haemorrhage, and improve the anticoagulant therapy by monitoring the efficacy and safety in the future.

In conclusion, we established a novel index, the MAT, which correlated with FXa activity. DBCM has the potential for evaluating FXa activity in addition to the global blood coagulability, and for monitoring the effect of anticoagulants including FXa inhibitors.

## Methods

### Study subjects

The study group consisted of a cumulative total of 74 healthy subjects. The healthy subjects were defined as having no medical history, no medications, no family history of coagulation deficiency, and no abnormal bleeding events. To study the basic characteristics of subjects, blood samples were analysed by LSI Medience (Tokyo, Japan). The study was approved by the ethics committee of Tokyo Medical and Dental University (No. M2000-1849). Blood samples were collected after written informed consent, and all experiments were performed in accordance with institutional regulation.

### Blood collection and preparation of anticoagulated samples

Blood samples were drawn from the cubital vein with minimum stasis. The first 0.5 ml of the drawn blood was discarded, and the remaining blood was kept in a tube containing 3.13% sodium citrate.

In this study, we used 3 anticoagulants with different FXa selectivity: unfractionated heparin (UFH), low molecular weight heparin (LMWH), and factor Xa inhibitor (FXaI). We utilized UFH (Mochida Pharmaceutical, Tokyo, Japan), and dalteparin (Pfizer, New York, NY, USA) as LMWH. Edoxaban was kindly supplied by Daiichi-Sankyo Corporation (Tokyo, Japan) and utilized as FXaI.

UFH or LMWH was added to the citrated blood samples to achieve final concentrations of 0, 0.05, 0.10, and 0.15 U/ml or IU/ml (Japanese Pharmacopoeia). Edoxaban was solved by 100% dimethyl sulfoxide to make an 180 mM stock solution, diluted with distilled water, and added to the blood samples to obtain final concentrations of 0, 100, 200, and 400 ng/ml. For the comparison to the anti-FXa assay, UFH and LMWH was added to achieve final concentration of 0, 0.075, and 0.15 U/ml or IU/ml, and edoxaban was added to obtain final concentrations of 0, 200, 400 ng/ml.

Whole blood was analysed by the DBCM, and plasma was used for the FXa activity assay, anti-FXa activity assay, and conventional coagulation assays. The plasma samples were obtained by centrifugation with 1500 × g for 15 minutes. The temperature for centrifugation was 23 °C for APTT measurement, and 4 °C for FXa assay and anti-FXa assay. The plasma samples were stored immediately at −30 °C or −80 °C until the measurements.

### Dielectric blood coagulometry

DBCM analyses were performed using a dielectric coagulometer (Sony Corp., Tokyo, Japan) as previously described^9^. This device was a prototype before approval for medical uses. Briefly, DBCM utilized 180 μl of citrated whole blood and the blood sample was initially mixed with 15 μl of 160 mM CaCl_2_. A blood sample was heated at 37 °C throughout the measurement. The DBCM measured the dielectric permittivity in a frequency range from 100 Hz to 16 MHz, with a sampling interval of 1 minute (Fig. 1). The dielectric permittivity was normalized compared to its initial value, and represented as normalized permittivity (Fig. 2a). The result of the DBCM was analysed by conducting a 5-point derivative of the dielectric permittivity at 10 MHz^9^ (Fig. 2b,c).

### FXa activity assay, anti-FXa activity assay, and activated partial thromboplastin time

The FXa activity was analysed using a fluorometric FXa assay kit (SensoLyte® Rh110 FXa Assay Kit, Anaspec, Fremont, CA, USA), and the fluorescent signal was measured using ARVO X5 (Perkin Elmer, Waltham, MA, USA). The APTT was measured by CA-50 (Sysmex, Kobe, Japan) using a Thrombocheck APTT-SLA (Sysmex).

The anti-FXa activity was analysed using a choromogenic anti-FXa assay kit (BIOPHEN HEPARIN (AT+), HYPHEN BioMed, France), and the absorbance was measured using iMark^TM^ Microplate Absorbance Reader (BIO-RAD, Herculus, CA, USA), according to the instruction from manufacturers.

### Study protocol

At first, we conducted the study to determine an adequate index to represent the FXa activity. Thirty healthy subjects were enrolled in this protocol. Citrated whole blood was used for the DBCM analysis, and plasma was utilized for the fluorometric FXa activity assay. The derivative of the dielectric permittivity was calculated as described previously^9^. We calculated the time to threshold by scanning the threshold values as a stepped proportion of the highest value of the derivative curve (Fig. 3). Each calculated time to threshold was evaluated by a correlation analysis with the FXa activity.

Next, we studied whether the DBCM analysis had a different response to different anticoagulants, focusing on their selectivity for FXa over thrombin. The blood samples were obtained and mixed with serial concentrations of UFH (n = 12), LMWH (n = 12), and FXaI (n = 20). The DBCM analysis was performed using whole blood. The APTT was also measured using plasma from 10 subjects of each anticoagulant group. For the comparison between the anti-FXa activity and DBCM analysis, 10 healthy subjects were enrolled.

### Statistical analysis

Statistical analyses were performed with JMP^®^10 software (SAS Institute Inc., Cary, NC, USA). Data are expressed as mean ± standard deviation. Single regression analyses were performed between 2 continuous variables. Correlations were assessed by Pearson correlation test. A p < 0.05 was considered statistically significant.

## Electronic supplementary material

Supplementary Information

## Data Availability

The datasets of the current study are available from the corresponding author on reasonable request.
