# Author Correction: Differential Assessment of Factor Xa Activity and Global Blood Coagulability Utilizing Novel Dielectric Coagulometry

**DOI:** 10.1038/s41598-018-37136-y

**Published:** 2019-03-15

**Authors:** Satomi Hamada, Yuki Hasegawa, Ai Oono, Anna Suzuki, Naomi Takahashi, Takuro Nishimura, Takatoshi Koyama, Michio Hagihara, Shuji Tohda, Tetsushi Furukawa, Kenzo Hirao, Tetsuo Sasano

**Affiliations:** 1 Department of Biofunctional Informatics, Tokyo Medical and Dental University (TMDU), Tokyo, Japan; 2 Department of Clinical Laboratory, Medical Hospital, Tokyo Medical and Dental University (TMDU), Tokyo, Japan; 3 Heart Rhythm Centre, Tokyo Medical and Dental University (TMDU), Tokyo, Japan; 4 Department of Laboratory Molecular Genetics of Haematology, Tokyo Medical and Dental University (TMDU), Tokyo, Japan; 5 Department of Bio-informational Pharmacology, Medical Research Institute, Tokyo Medical and Dental University (TMDU), Tokyo, Japan; 6 Department of Cardiovascular Medicine, Tokyo Medical and Dental University (TMDU), Tokyo, Japan; 7 Department of Cardiovascular Physiology, Tokyo Medical and Dental University (TMDU), Tokyo, Japan

Correction to: *Scientific Reports* 10.1038/s41598-018-34229-6, published online 31 October 2018

In this Article the Competing Interests section was omitted. This should read:

“T.S. received research funding from Sony IP&S Inc.”

