# Supplementary material for: Differential Assessment of Factor Xa Activity and Global Blood Coagulability Utilizing Novel Dielectric Coagulometry
Source: Sci Rep. 2018 Oct 31;8:16129. doi: 10.1038/s41598-018-34229-6 (PMC6208345; doi:10.1038/s41598-018-34229-6)
Supplement: Supplementary file 1 — Supplementary Information [file 41598_2018_34229_MOESM1_ESM.pdf]

Supplementary Information

Differential Assessment of Factor Xa Activity and Global Blood Coagulability Utilizing Novel Dielectric Coagulometry

Satomi Hamada, Yuki Hasegawa, Ai Oono, Anna Suzuki, Naomi Takahashi, Takuro Nishimura, Takatoshi Koyama, Michio Hagihara, Shuji Tohda, Tetsushi Furukawa, Kenzo Hirao, Tetsuo Sasano

Supplementary Table S1
Basic characteristics of the study subjects

		Value
Age	(years)	28 ± 10
Male sex	n,(%)	5 (50)
White blood cell	($\times 10^9$/l)	5.4 ± 0.9
Red blood cell	($\times 10^{12}$/l)	4.55 ± 0.44
Hemoglobin	(g/l)	137 ± 13
Hematocrit	(l/l)	0.427 ± 0.041
Platelet	($\times 10^9$/l)	262 ± 45
Thrombin-antithrombin complex*	(ng/ml)	1.4 ± 0.5
Total protein	(g/dl)	7 ± 0.5
Albumin	(g/dl)	4.6 ± 0.2
Aspartate aminotransferase	(U/l)	18 ± 9
Alanine aminotransferase	(U/l)	13 ± 6
Lactate dehydrogenase	(U/l)	154 ± 37
Creatinine	(mg/dl)	0.72 ± 0.18
Urea nitrogen	(mg/dl)	12.8 ± 3.2
C-reactive protein	(mg/dl)	0.02 ± 0.02

N = 10. *: One sample was excluded due to handling error of sample. Data are expressed as mean ± standard deviation except for male sex.

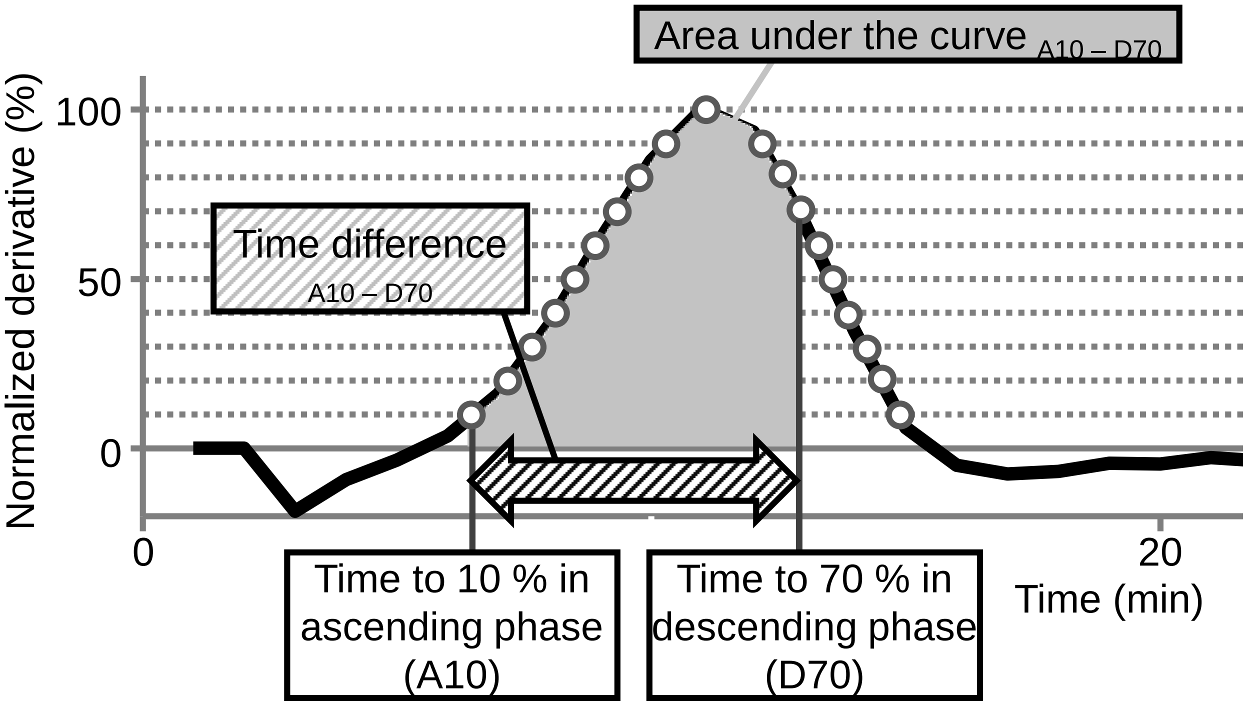

Supplementary Figure S1

Definition of additional DBCM parameters

We scanned the threshold line from 10 % to 90 % of the maximum value of the derivative (dotted line). When the threshold line was set by *n* % of the maximum value in ascending phase, time to threshold is named as A*n.* When the threshold line was set by *n* % of the maximum value in descending phase, time to threshold is named as D*n*. Definition of A10 and D70 are indicated in Figure as examples.

Using these times to threshold, we defined 2 additional parameters. Time difference is a difference between 2 time points (ex. Time $\text{difference}_{\text{A10-D70}}$ indicates a time difference between A10 and D70). The area under the curve (AUC) is an integral of the value between 2 time points (ex. Area under the $\text{curve}_{\text{A10-D70}}$ indicates an AUC between A10 and D70).

Supplementary Table S2
Correlations between other DBCM parameters and factor Xa activity

DBCM parameter	r	p
Time difference		
A10 – A20	-0.021	0.913
A10 – A30	-0.048	0.800
A10 – A40	-0.227	0.227
A10 – A50	-0.177	0.350
A10 – A60	-0.185	0.328
A10 – A70	-0.231	0.220
A10 – A80	-0.244	0.195
A10 – A90	-0.191	0.312
A10 – MAT	-0.309	0.097
A10 – D90	-0.200	0.290
A10 – D80	-0.230	0.223
A10 – D70	-0.204	0.278
A10 – D60	-0.217	0.248
A10 – D50	-0.198	0.295
A10 – D40	-0.213	0.259
A10 – D30	-0.182	0.336
A10 – D20	-0.184	0.331
A10 – EAT	-0.184	0.330
A20 – EAT	-0.180	0.340
A30 – EAT	-0.179	0.345
A40 – EAT	-0.111	0.559
A50 – EAT	-0.133	0.483
A60 – EAT	-0.123	0.517
A70 – EAT	-0.106	0.576
A80 – EAT	-0.089	0.640
A90 – EAT	-0.122	0.521
MAT – EAT	-0.047	0.806
D90 – EAT	-0.102	0.592
D80 – EAT	-0.079	0.676
D70 – EAT	-0.096	0.613
D60 – EAT	-0.064	0.736
D50 – EAT	-0.093	0.626
D40 – EAT	-0.003	0.988
D30 – EAT	-0.094	0.622
D20 – EAT	-0.065	0.734

DBCM parameter	r	p
Area under the curve		
A10 – A20	0.108	0.568
A10 – A30	0.148	0.436
A10 – A40	-0.280	0.134
A10 – A50	-0.123	0.516
A10 – A60	-0.137	0.471
A10 – A70	-0.252	0.179
A10 – A80	-0.287	0.124
A10 – A90	-0.111	0.558
A10 – MAT	-0.327	0.078
A10 – D90	-0.142	0.454
A10 – D80	-0.171	0.367
A10 – D70	-0.148	0.434
A10 – D60	-0.165	0.382
A10 – D50	-0.150	0.428
A10 – D40	-0.163	0.390
A10 – D30	-0.148	0.434
A10 – D20	-0.149	0.433
A10 – EAT	-0.152	0.422
A20 – EAT	-0.149	0.432
A30 – EAT	-0.151	0.426
A40 – EAT	-0.116	0.541
A50 – EAT	-0.133	0.485
A60 – EAT	-0.123	0.518
A70 – EAT	-0.107	0.573
A80 – EAT	-0.090	0.636
A90 – EAT	-0.131	0.490
MAT – EAT	-0.021	0.914
D90 – EAT	-0.114	0.549
D80 – EAT	-0.076	0.691
D70 – EAT	-0.121	0.523
D60 – EAT	-0.044	0.818
D50 – EAT	-0.089	0.640
D40 – EAT	0.136	0.473
D30 – EAT	-0.082	0.665
D20 – EAT	-0.067	0.727

DBCM, dielectric blood coagulometry; MAT, maximum acceleration time; EAT, end of acceleration time.

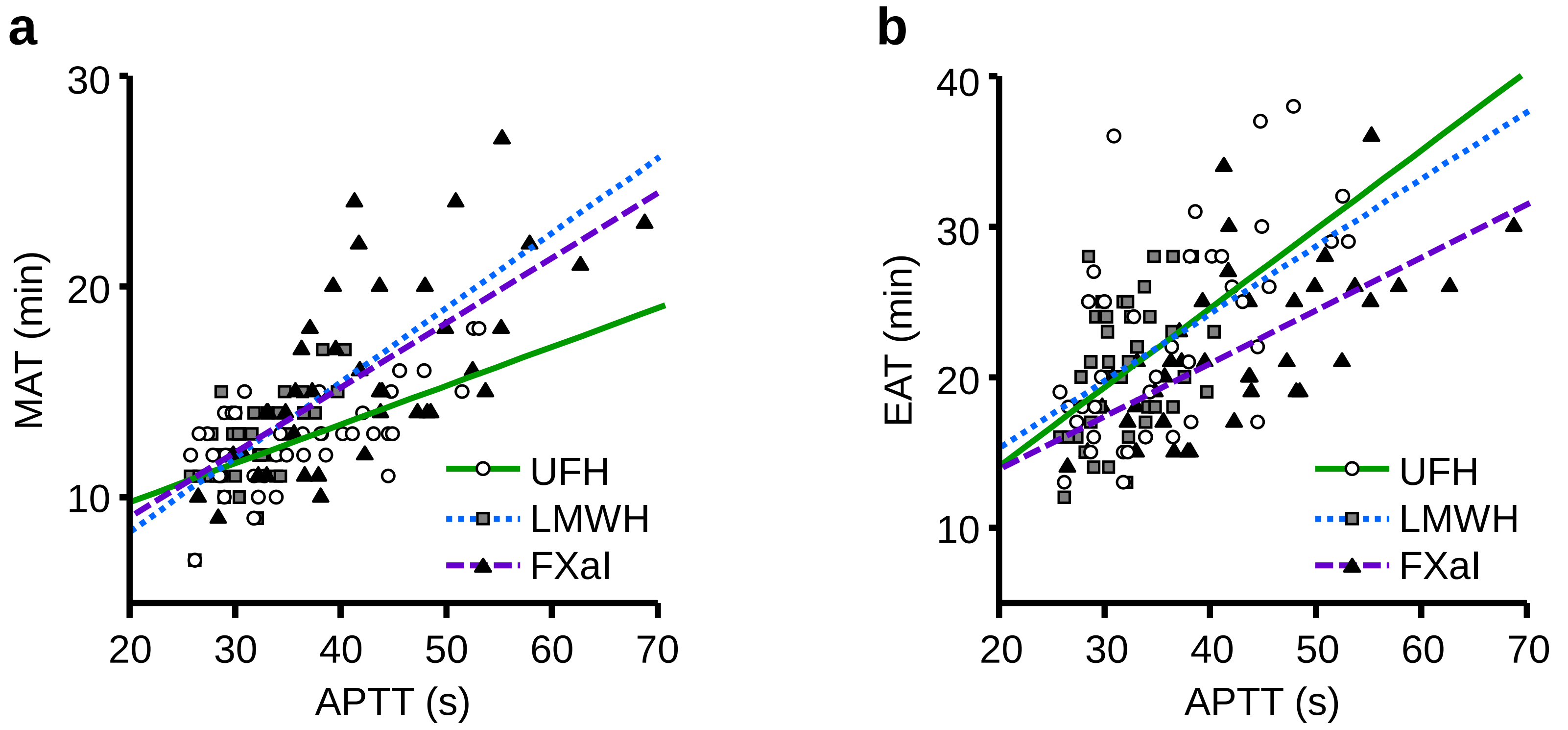

Supplementary Figure S2

Correlation between the APTT and DBCM parameters

The relationship between the APTT and the MAT (a) or EAT (b) is plotted, and single regression analyses are performed. APTT, activated partial thrombin time; DBCM, dielectric blood coagulometry; MAT, maximum acceleration time; EAT, end of acceleration time; UFH, unfractionated heparin; LMWH, low molecular weight heparin; FXaI, factor Xa inhibitor.
